# Effect of 5-aza-2ˈ-deoxycytidine on *p27Kip1*, *p21Cip1/Waf1/Sdi1*, *p57Kip2*, and *DNA methyltransferase 1* Genes Expression, Cell Growth Inhibition and Apoptosis Induction in Colon Cancer SW 480 and SW 948 Cell Lines

**DOI:** 10.31661/gmj.v9i0.1899

**Published:** 2020-12-26

**Authors:** Masumeh Sanaei, Fraidoon Kavoosi, Sedighe Nasiri

**Affiliations:** ^1^Research Center for Non-communicable Diseases, Jahrom University of Medical Sciences, Jahrom, Iran; ^2^Student of Research Committee, Jahrom University of Medical Sciences, Jahrom, Iran

**Keywords:** 5-aza-CdR, Cip/Kip Genes, DNMT1, Colon Cancer

## Abstract

**Background:**

Dysregulation of the cell cycle has been reported in various cancers. Inactivation of the cyclin-dependent kinases inhibitors (CDKIs), CIP/KIP family, such as *p21Cip1/Waf1/Sdi1, p27Kip1,* and *p57Kip2* genes because of hypermethylation has been shown in several cancers. Treatment with DNA demethylating agent 5-aza-2ˈ-deoxycytidine (5-Aza-CdR) has been indicated that affect genomic methylation and resulting in silenced genes reactivation in colon cancer. Previously, we evaluated the effect of 5-Aza-CdR on *DNA methyltransferase 1* (*DNMT1*) gene expression in hepatocellular carcinoma (HCC) which encouraged us to design the current study. The present study aimed to evaluate the effect of 5-Aza-CdR on *p21Cip1/Waf1/Sdi1, p27Kip1, p57Kip2*, and *DNAT1* genes expression, cell growth inhibition and apoptosis induction in colon cancer SW 480 and SW 948 cell lines.

**Materials and Methods:**

The effect of 5-aza-CdR on the SW 480 and SW 948 cells growth, apoptosis induction and genes expression were assessed by MTT assay, flow cytometry, and real-time quantitative reverse transcription-polymerase chain reaction (qRT-PCR) analysis respectively.

**Results:**

5-aza-CdR inhibited cell growth as time-and dose-dependent manner significantly (P<0.001). The agent reactivated *p15INK4, p16INK4, p18INK4,* and *p19INK4* genes expression and induced apoptosis at a concentration of 5 μM significantly. Besides, 5-aza-CdR had a more significant effect on the SW 480 cell line in comparison to SW 948 cell line.

**Conclusion:**

5-Aza-CdR plays a key role in the up-regulation of *p21Cip1/ Waf1/Sdi1, p27Kip1,* and *p57Kip2* and down-regulation of DNMT1 genes resulting in cell growth inhibition and apoptosis induction.

## Introduction

Cancer is considered to be a disease of the cell cycle deregulation, the most frequent alterations during cancer induction and tumor development which underlies the aberrant cell proliferation. Cell cycle progression is a highly ordered, tightly-regulated process that involves several checkpoints that evaluate extracellular cell growth signals, cell size, and DNA integrity [1]. The cell cycle progression is driven by the cyclin-dependent kinase (CDK) family, the key regulators of cell-cycle transitions, and their regulatory partners, the cyclins. The level of cyclins fluctuates throughout the cell cycle. In mammalian cells, G1 phase progression is driven by cyclin *D-CDK4, cyclin D-CDK6*, and * cyclin E-CDK2. Cyclin A-CDK2* involve in S phase initiation and *cyclin B-CDK1* regulates G2 phase progression [2]. Both the cyclin D and E and their associated kinases are necessary for entry into and progression through the G1 phase of the cell cycle. The *CDKs* family are the major targets for deregulation in cancer and the misregulated *CDKs* lead to unscheduled cell proliferation as well as chromosomal and genomic instability [3]. Inhibition of cyclin-dependent kinases activity may be effective anti-cancer therapeutics. These kinases can be negatively regulated by two groups of cyclin-dependent kinases inhibitors (CDKIs) including INK4 and Cip/Kip families. INK4 proteins, specific for the *Cdk4 *subfamily, include *p16INK4a, p15INK4b, p18INK4c,* and *p19INK4d* and interact with the monomeric *CDKs*. The Cip/Kip family includes *p21Cip1, p27Kip1, *and *p57Kip2 *which contact both the CDK and cyclin subunits and can inhibit CDK-cyclin heterodimers [4]. DNA methylation can affect chromatin structure, which, in turn, can alter tumor suppressor genes (TSGs) expression. These changes have been involved in tumorigenesis. Hypermethylation of CDKIs has been reported in various cancers such as colon cancer [5]. DNA methylation is brought by a family of enzymes known as the DNA methyltransferases (DNMTs) including DNMT1, DNMT1b, DNMT1o, DNMT1p, DNMT2, DNMT3a, DNMT3b with its isoforms, and DNMT3L [6]. Treatment with DNA demethylating agent 5-aza-2ˈ-deoxycytidine (5-Aza-CdR) has been indicated that affect genomic methylation and resulting in silenced genes reactivation in colon cancer [7,8]. In addition to colon cancer, it has been reported that 5-aza-CdR increases *p53/p21Waf1/Cip1* expression in lung cancer and prostate cancer [9,10], *p27kip1* in esophageal squamous cell carcinoma [11], and *p57KIP2 *in lung and breast cancers [12]. Previously, we evaluated the effect of 5-Aza-CdR on *DNMT1* gene expression in hepatocellular carcinoma (HCC) which encouraged us to design the current study [13]. The present study aimed to evaluate the effect of 5-Aza-CdR on *p21Cip1/Waf1/Sdi1, p27Kip1, p57Kip2, and DNA methyltransferase 1*genes expression, cell growth inhibition and cell apoptosis induction in colon cancer SW 480 and SW 948 cell lines.

## Materials and Methods

 Human colon cancer SW 480 and SW 948 cell lines were purchased from the National Cell Bank of Iran-Pasteur Institute and cultured and maintained in Dulbecco’s modified Eagle’s medium (DMEM) supplemented with fetal bovine serum 10% and antibiotics in a humidified atmosphere of 5% CO2 in air at 37°C. 5-aza-CdR was provided from Sigma (St. Louis, MO, USA) and dissolved in dimethyl sulfoxide (DMSO; Sigma) at a final concentration of 100 μM to obtain a stock solution. All other working solutions were provided by diluting the stock solution. Antibiotics, DMSO, trypsin-EDTA, 3-[4, 5-dimethyl-2-thiazolyl]-2, 5-diphenyl-2H-tetrazolium bromide (MTT), Annexin-V-(FITC), propidium iodide (PI), DMEM, and Phosphate-buffered saline (PBS) were purchased from Sigma. Total RNA extraction kit (TRIZOL reagent) and real-time polymerase chain reaction (PCR) kits (qPCR MasterMix Plus for SYBR Green I dNTP) were obtained from Applied Biosystems Inc. (Foster, CA, USA). This work was approved in the Ethics Committee of Jahrom University of Medical science with a code number of IR.JUMS.REC.1397.138.

###  Cell Growth and Viability 

 The effect of 5-aza-CdR on the in vitro growth of colon cancer SW 480 and SW 948 cell lines was determined by MTT assay. Briefly, the cells were seeded at the density of 5 × 105 cells per well onto a 96-well plate for 24h. The cells were subsequently treated with 5-aza-CdR (0.5, 1, 2.5, 5, and 10 μM) for 24h and 48h. After treatment times, 20 μl of MTT (0.5%) in PBS was added to each well and the incubation was continued for 4 h at 37˚C and then the culture medium was replaced with DMSO (200 μl ) and finally, the optical density was detected by a microplate reader at a wavelength of 570 nM. Each sample was performed in triplicate.

###  Flow Cytometry Analysis

 The percentage of colon cancer SW 480 and SW 948 apoptotic cells was evaluated by staining the cells with annexin V-FITC, a sensitive probe for identifying cells undergoing apoptosis in an early stage which precedes the loss of membrane integrity, and PI, allowing to distinguish annexin V-single positive cells undergoing the late apoptosis according to the manufacturer’s protocol. Before flow cytometry analysis, the cells were plated at a density of 5 × 105 per well in 24-well plate and treated with 5-aza-CdR (1 μM) for different periods (24 and 48 h) except for the control groups, these groups were incubated with DMSO only. Subsequently, all adherent and floating cells were collected by trypsinization and washed with cold PBS and resuspended in binding buffer for 10 min at room temperature in the dark. Finally, the cells were incubated with annexin-V-(FITC) and PI according to the manufacturer’s protocol and the double-stained cells were subsequently analyzed by FACScan flow cytometry (Becton Dickinson, Heidelberg, Germany). Three independent experiments were performed for each concentration.

###  Real-Time Quantitative Reverse Transcription-Polymerase Chain Reaction

###  ( qRT -PCR) Analysis

Total RNA was extracted from 5-aza-CdR (1μM) treated colon cancer cell, SW 480 and SW 948, using TRIzol reagent (Invitrogen, Carlsbad, CA, USA) and treated by RNase-free DNase (Qiagen). Subsequently, One microgram RNA was reverse transcribed using the QuantiTect Reverse Transcription Kit (Qiagen, Hilden, Germany) to generate the single-stranded cDNAs. The expression of *p21Cip1/Waf1/Sdi1, p27Kip1, p57Kip2, *and *DNMT1* genes were quantified using the quantitative SYBR Green PCR kit (TaKaRa Bio, Japan) according to the manufacturer’s protocol and measured by quantitative real-time PCR using StepOnePlus (Applied Biosystem, USA) instrument. Primers sequences of the mentioned genes are indicated in Table-1. GAPDH was used as an endogenous control. Data were analyzed using the comparative Ct (ΔΔct) method. The thermocycling condition was as described previously [14].

## Results

###  Result of Cell Viability by the MTT Assay 

**Figure 1:**
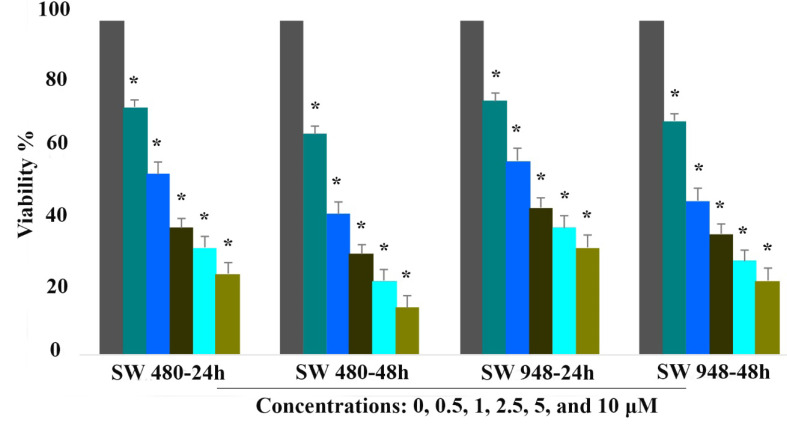
The effect of 5-aza-CdR on colon cancer SW 480 and SW 948 cells viability. The cells were with different concentrations of 5-aza-CdR (0.5, 1, 2.5, 5, and 10 μM) for 24 and 48 h and then the cell viability was evaluated by MTT assay. Each experiment was done in triplicate Asterisks (*) indicate significant differences between treated and untreated cells.

 To examine the inhibitory effect of 5-aza-CdR on colon cancer SW 480 and SW 948 cells, we treated the cells with various concentrations of the agent (0.5, 1, 2.5, 5, and 10 μM) for 24 and 48h. The viability of SW 480 and SW 948 cells was determined by MTT assay. As shown in Figure-1, 5-aza-CdR inhibited cell growth significantly in a time- and dose-dependent manner (P<0.001). The IC50 value was obtained with approximately 1 μM of 5-aza-CdR.

###  Result of Cell Apoptosis Assay

**Figure 2:**
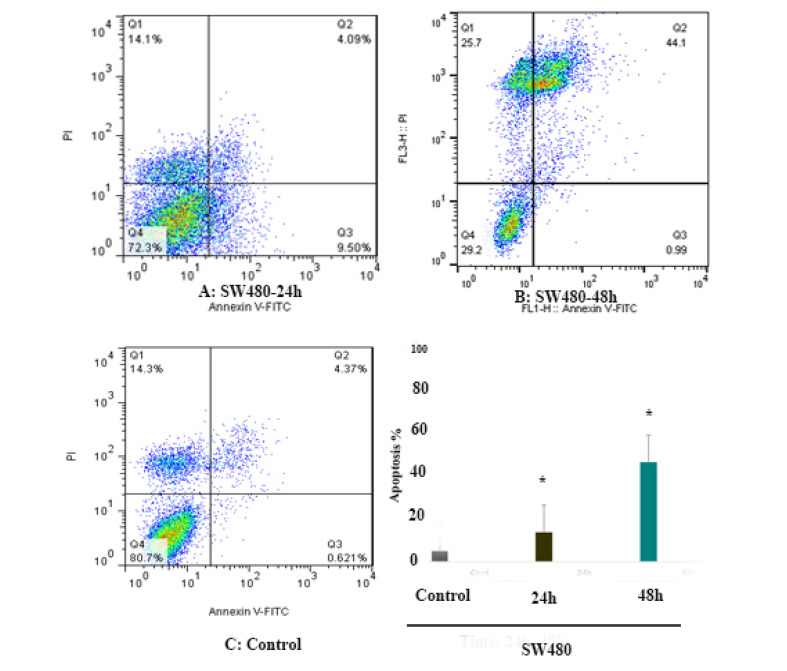
The apoptosis-inducing effect of 5-aza-CdR on colon cancer SW 480 cells investigated by flow cytometry assay. As shown above, 5-aza-CdR induced apoptosis as a time-dependent manner. Asterisks (*) indicate significant differences between treated and untreated cells.

**Figure 3:**
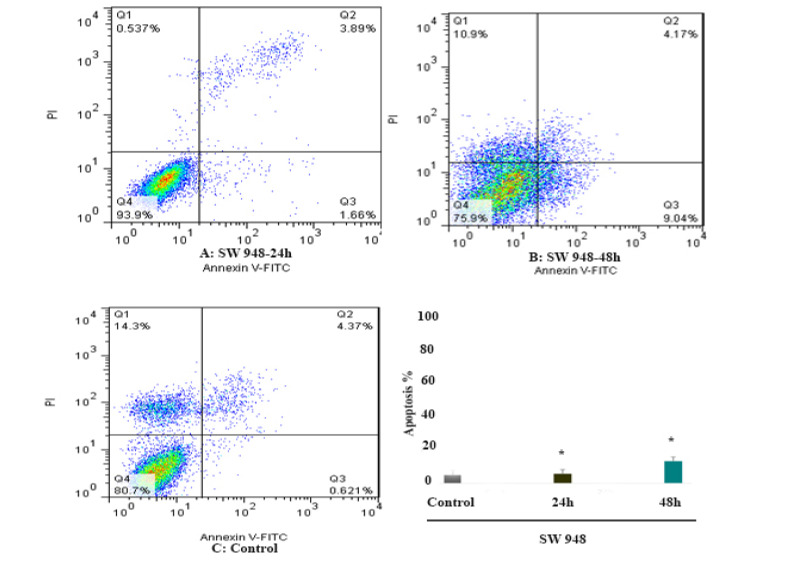
The apoptosis-inducing effect of 5-aza-CdR on colon cancer SW 948 cells investigated by flow cytometry assay. As shown above, 5-aza-CdR induced apoptosis as a time-dependent manner. Asterisks (*) indicate significant differences between treated and untreated cells.

**Figure 4:**
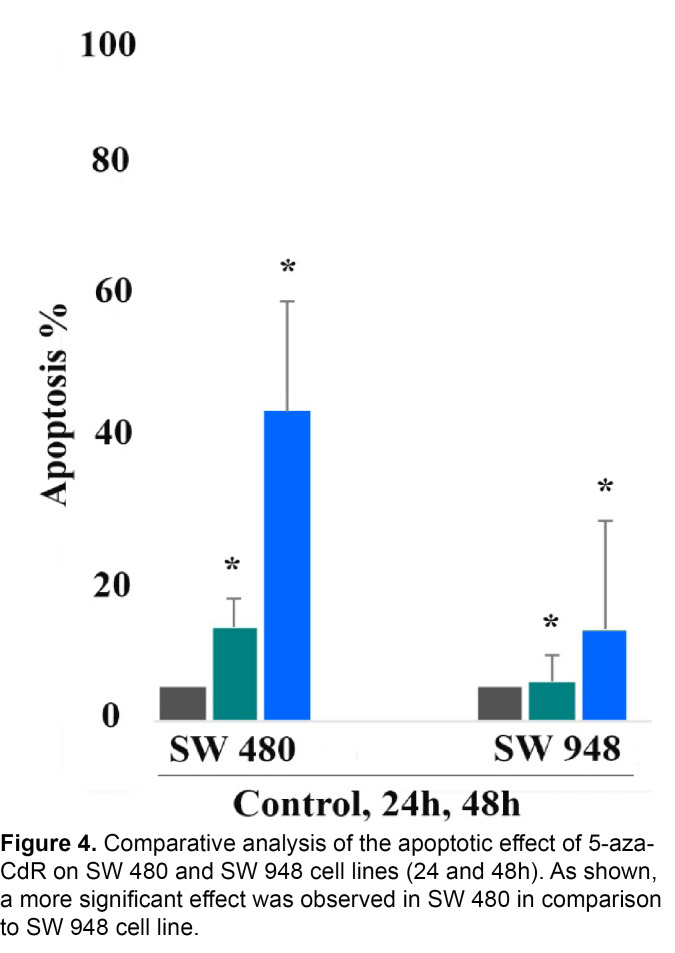
Comparative analysis of the apoptotic effect of 5-aza-CdR on SW 480 and SW 948 cell lines (24 and 48h). As shown, a more significant effect was observed in SW 480 in comparison to SW 948 cell line.

 Cell apoptosis was measured by Annexin V and PI staining. To determine the effect of 5-aza-CdR (1 μM) on SW 480 and SW 948 cells apoptosis, the cells were stained using annexin-V-(FITC) to detect the apoptotic cells in an early stage and PI to detect annexin V-single positive cells from the cells subjected to necrotic processes. After treatment times (24 and 48 h), the treated and untreated cells were collected by trypsinization and labeled with annexin V and PI. Representative graphs are indicated in Figures-2 and 3. As shown, significant differences were observed between the numbers of apoptotic cells in SW 480 treated groups compared to untreated control groups, Figure-2. In SW 948 cell line, the significant apoptotic effect was observed after 48 h of treatment as indicated in Figure-3. As indicated in Figure-4, 5-aza-CdR had a more significant apoptotic effect on the SW 480 cell line in comparison to SW 948 cell line. The percentage of the apoptotic cells is shown in Table-2.

###  Result of Determination of Genes Expression

**Figure 5:**
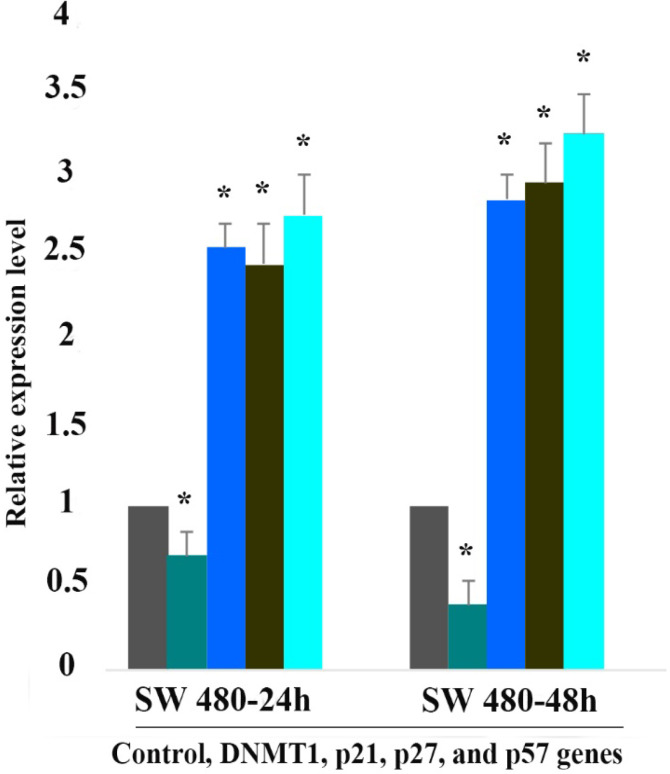
The relative expression level of p21Cip1/Waf1/Sdi1, p27Kip1, p57Kip2 and DNMT1 genes treated with 5-aza-CdR in SW 480 cells. Significant up-regulation of p21Cip1/Waf1/Sdi1, p27Kip1, and p57Kip2 and down-regulation of DNMT1 genes was observed after treatment with the compound at different times (24 and 48h). Asterisks (*) indicate significant differences between treated and control groups. Data are presented as the means ± standard error of the mean.

**Figure 6:**
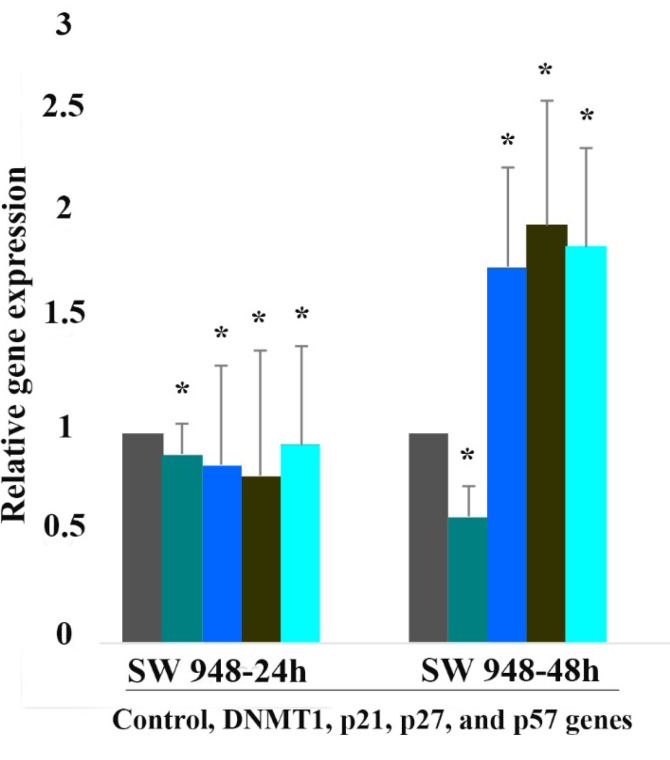
The relative expression level of p21Cip1/Waf1/Sdi1, p27Kip1, p57Kip2 and DNMT1 genes treated with 5-aza-CdR in SW 948 cells. Significant up-regulation of p21Cip1/Waf1/Sdi1, p27Kip1, and p57Kip2 and down-regulation of DNMT1 genes was observed after treatment with the compound for 48h. No significant effect was observed after 24h of treatment. Asterisks (*) indicate significant differences between treated and control groups. Data are presented as the means ± standard error of the mean.

The effect of 5-aza-CdR (1 μM) on *p21Cip1/Waf1/Sdi1, p27Kip1, p57Kip2, *and * DNMT1* genes expression was evaluated by quantitative real-time RT-PCR analysis. The result of RT-PCR analysis revealed that treatment with 5-aza-CdR for 24 and 48 h up-regulated *p21Cip1/Waf1/Sdi1, p27Kip1, *and *p57Kip2* and down-regulated DNMT1 genes significantly in SW 480 cells (Figure-5). No significant effect was observed in SW 948 after 24h of treatment but treatment with 5-aza-CdR for 48h induced significant up-regulation of *p21Cip1/Waf1/Sdi1, p27Kip1, *and * p57Kip2* and down-regulation of *DNMT1* genes (Figure-6). The relative expression of all of the genes has been shown in Table-3.

## Discussion

Dysregulation of the cell cycle has been reported in various cancers. Inactivation of the CIP/KIP family such as *p21, p27, *and * p57* genes because of hypermethylation has been shown in several cancers including myelodysplastic syndrome (MDS), acute myeloid leukemia (AML) [18,19], and colorectal cancers [20]. DNA demethylating agents such as 5-aza-CdR can reactive hypermethylated cyclin-dependent kinase inhibitors in hepatocellular cancer Hep G2 [21], human ovarian cancer SKOV3 cells [22], pancreatic cancer [23], and colon cancer [24]. In this study, we indicated that 5-aza-CdR inhibits cell growth and induces apoptosis in SW 480 and SW 948 cell lines as a time- and dose-dependent manner. To evaluate the mechanisms involved in cell apoptosis following treatment with 5-aza-CdR, we investigated the genes expression and found that this agent plays its role through the up-regulation of *p21Cip1/Waf1/Sdi1, p27Kip1,* and * p57Kip2* and down-regulation of DNMT1 genes expression. Besides, we demonstrated that 5-aza-CdR had a more significant effect on the SW 480 cell line in comparison to SW 948 cell line. Similar pathways have been reported in other cancers. In HCC SMMC-7721 cell lines, 5-aza-CdR can reactivate *p15, p16, p21* genes by DNA demethylation [25]. In prostate cancer PC3, LNCaP and DU145 cell lines, the demethylating agent 5-Aza-CdR can up-regulate *p21WAF1/CIP1* mRNA expression [26]. The demethylation of *p27kip1* cells treated with 5-Aza-CdR has been demonstrated in the gastric cancer cell line too [27]. Other researchers have been shown that 5-Aza-CdR can restore methylated *p57KIP2* in lung and breast cancer cell lines [28]. It has been established that deregulation in the function of CDK inhibitors can result in tumorigenesis processes. There are two families of CDK-inhibitors: INK4 and CIP/KIP class. These two families differ in the particular cyclin families that they interact with. The kinase inhibitor family, CIP/KIP, is composed of three proteins that interact with other cyclin families. From a mechanistic standpoint, CDK-inhibitors can be used as an anti-cancer drug by blocking CDK’s [29]. As we reported in the current article, others have indicated a decreased expression of DNMT1 has been reported following treatment with 5-Aza-CdR in colorectal cancer HCT 116, HT-29, MIP101, and RKO cell lines [30]. Furthermore, it has been indicated that 5-Aza-CdR demethylates the promoter sequence of *TIMP-3 *and * p16* in HCT116 colon cancer [31]. Additionally, 5-Azadc induce the 15-lipoxygenase-1 (15-LOX-1) expression in human colon cancer cells which increases 13-S-hydroxyoctadecadienoic acid levels, cell growth inhibition, and apoptosis induction in these cells [32]. All reports mentioned above are inconsistent with our results. In addition to Cyp/Kip pathway and DNMT1 inhibition which reported by our teamwork, other molecular mechanisms have been reported for 5-Aza-CdR. It induces apoptosis by up-regulation of human and mouse *TNFR1* (TNFRSF1) genes [33]. Another study has been demonstrated that this agent restores the expression of TBX5 in colon cancer cell lines SW620, HT-29, SW620 and CaCO2 [34]. In gastrointestinal cancers, it can induce apoptosis by re-activation of silenced death-associated protein kinase expression [35]. Finally, the up-regulation of Cip/Kip genes and down-regulation of DNMT1 gene is not the only molecular pathways of apoptosis in colon cancer. The following of other mechanisms is recommended.

## Conclusion

In summary, we have demonstrated that 5-Aza-CdR plays a key role in re-activation of *p21Cip1/Waf1/Sdi1, p27Kip1, *and * p57Kip2* and down-regulation of DNMT1 genes resulting in cell growth inhibition and apoptosis induction. This pathway may be an effective molecular target for colon cancer treatment through the re-activation of Cip/Kip and inhibition of *DNMT1 *genes.

## Acknowledgment

 This article was supported by the adjutancy of research of Jahrom University of Medical Sciences, Iran. The article has been extracted from Ms. Sedighe Nasiri’s thesis.

## Conflict of Interest

 The authors report no conflict of interest.

**Table 1:** The Primer Sequences of *p21Cip1/Waf1/Sdi1*, p27Kip1, * p57Kip2*, and *DNMT1* Genes.

**Primer name**		**Primer sequences (5’ to 3’)**	References
***DNMT1***	**Forward**	GAG GAA GCT GCT AAG GAC TAG TTC	[15]
	**Reverse**	ACT CCA CAA TTT GAT CAC TAA ATC	
***P21***	**Forward**	AGG CGC CAT GTC AGA ACC GGC TGG	[16]
	**Reverse**	GGA AGG TAG AGC TTG GGC AGG C	
***P 27***	**Forward**	ATG TCA AAC GTG CGA GTG TCT AAC	[16]
	**Reverse**	TTA CGT TTG ACG TCT TCT GAG GCC A	
***P 57***	**Forward**	GCGGCGATCAAGAAGCTGTC	[17]
	**Reverse**	CCGGTTGCTGCTACATGAAC	
***GAPDH***	**Forward**	TCCCATCACCATCTTCCA	[17]
	**Reverse**	CATCACGCCACAGTTTCC	

**Table 2:** The Percentage of Apoptotic Cells Treated with 5-Aza-CdR at Different Time Periods.

**Cell line**	**Drug**	**Dose ( μ M )**	**Duration (h)**	**Apoptosis (%)**	**P-value**
**SW 480 **	5-Aza-CdR	5	24	13.59	0.001
**SW 480**	5-Aza-CdR	5	48	45.09	0.001
**SW 948**	5-Aza-CdR	5	24	5.55	0.690
**SW 948**	5-Aza-CdR	5	48	13.21	0.001

**Table 3:** Relative Expression Level of *p21Cip1/Waf1/Sdi1*, * p27Kip1*, * p57Kip2* and *DNMT1* Genes.

**Cell line**	**Gene**	**Drug**	**Dose ( μM ) **	**Duration (h)**	**Expression**	**P-value**
**Sw 480**	*p21Cip1/Waf1/Sdi1*	5-Aza-CdR	5 μM	24	2.6	0.001
**Sw 480**	*p21Cip1/Waf1/Sdi1*	5-Aza-CdR	5 μM	48	2.9	0.001
**Sw 480**	*p27Kip1*	5-Aza-CdR	5 μM	24	2.5	0.001
**Sw 480**	*p27Kip1*	5-Aza-CdR	5 μM	48	3	0.001
**Sw 480**	*p57Kip2*	5-Aza-CdR	5 μM	24	2.8	0.001
**Sw 480**	*p57Kip2*	5-Aza-CdR	5 μM	48	3.3	0.001
**Sw 480**	*DNMT1*	5-Aza-CdR	5 μM	24	0.7	0.028
**Sw 480**	*DNMT1*	5-Aza-CdR	5 μM	48	0.4	0.001
**SW 948**	*p21Cip1/Waf1/Sdi1*	5-Aza-CdR	5 μM	24	0.85	0.70
**SW 948**	*p21Cip1/Waf1/Sdi1*	5-Aza-CdR	5 μM	48	1.8	0.001
**SW 948**	*p27Kip1*	5-Aza-CdR	5 μM	24	0.8	0.47
**SW 948**	*p27Kip1*	5-Aza-CdR	5 μM	48	2	0.001
**SW 948**	*p57Kip2*	5-Aza-CdR	5 μM	24	0.95	0.99
**SW 948**	*p57Kip2*	5-Aza-CdR	5 μM	48	1.9	0.001
**SW 948**	*DNMT1*	5-Aza-CdR	5 μM	24	0.9	0.90
**SW 948**	*DNMT1*	5-Aza-CdR	5 μM	48	0.6	0.001
